# Angiotensin II triggers release of neutrophil extracellular traps, linking thromboinflammation with essential hypertension

**DOI:** 10.1172/jci.insight.148668

**Published:** 2021-09-22

**Authors:** Akrivi Chrysanthopoulou, Eugenia Gkaliagkousi, Antonios Lazaridis, Stella Arelaki, Panagiotis Pateinakis, Maria Ntinopoulou, Alexandros Mitsios, Christina Antoniadou, Christos Argyriou, George S. Georgiadis, Vasileios Papadopoulos, Alexandra Giatromanolaki, Konstantinos Ritis, Panagiotis Skendros

**Affiliations:** 1 Laboratory of Molecular Hematology, Department of Medicine, Democritus University of Thrace, Alexandroupolis, Greece.; 2 Third Department of Internal Medicine, Papageorgiou General Hospital, Aristotle University of Thessaloniki, Thessaloniki, Greece.; 3 Translational Functional Cancer Genomics, National Center for Tumor Diseases and German Cancer Research Center, Heidelberg, Germany.; 4 Department of Nephrology, Papageorgiou General Hospital, Thessaloniki, Greece.; 5 First Department of Internal Medicine,; 6 Department of Vascular Surgery, and; 7 Department of Pathology, University Hospital of Alexandroupolis, Democritus University of Thrace, Alexandroupolis, Greece.

**Keywords:** Immunology, Inflammation, Hypertension, Neutrophils, Thrombin

## Abstract

Innate immunity and chronic inflammation are involved in atherosclerosis and atherothrombosis, leading to target organ damage in essential hypertension (EH). However, the role of neutrophils in EH is still elusive. We investigated the association between angiotensin II (Ang II) and neutrophil extracellular traps (NETs) in pathogenesis of EH. Plasma samples, kidney biopsies, and surgical specimens of abdominal aortic aneurysms (AAAs) from patients with EH were used. Cell-based assays, NETs/human aortic endothelial cell cocultures, and in situ studies were performed. Increased plasma levels of NETs and tissue factor (TF) activity were detected in untreated, newly diagnosed patients with EH. Stimulation of control neutrophils with plasma from patients with untreated EH generated TF-enriched NETs promoting endothelial collagen production. Ang II induced NETosis in vitro via an ROS/peptidylarginine deiminase type 4 and autophagy-dependent pathway. Circulating NETs and thrombin generation levels were reduced substantially in patients with EH starting treatment with Ang II receptor blockers, whereas their plasma was unable to trigger procoagulant NETs. Moreover, TF-bearing NETotic neutrophils/remnants accumulated in sites of interstitial renal fibrosis and in the subendothelial layer of AAAs. These data reveal the important pathogenic role of an Ang II/ROS/NET/TF axis in EH, linking thromboinflammation with endothelial dysfunction and fibrosis.

## Graphical abstract

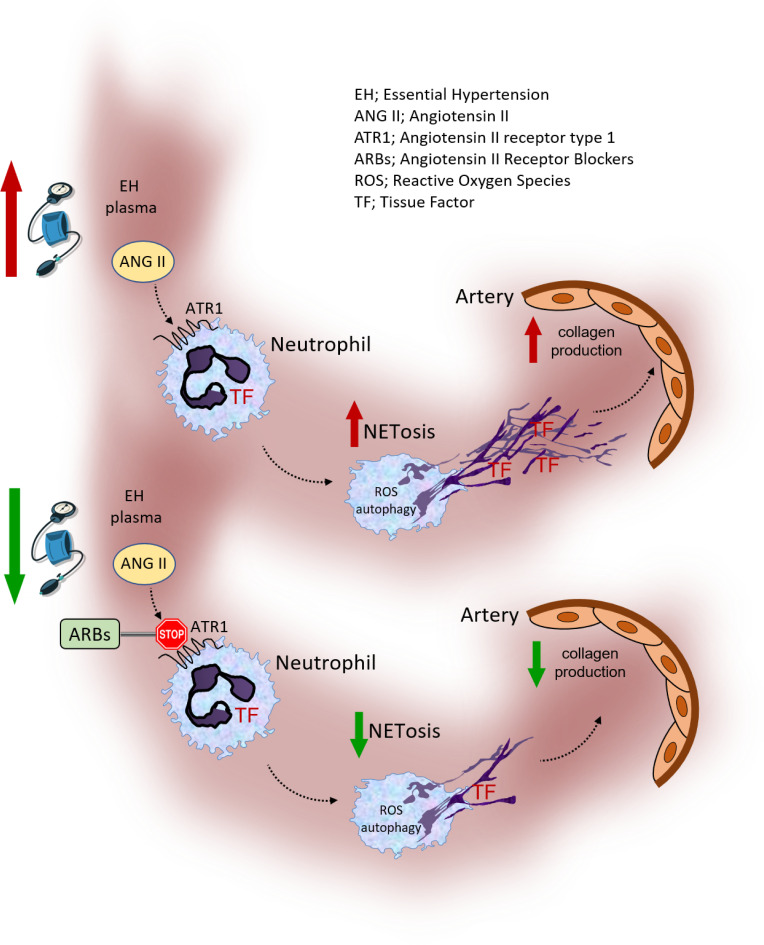

## Introduction

Essential hypertension (EH) is a major risk factor for cardiovascular disease (CVD) and chronic kidney disease (CKD), constituting a leading cause of morbidity and mortality (1, 2). Endothelial dysfunction, oxidative stress, and chronic low-grade inflammation have been documented to contribute to the initiation and maintenance of EH (3), leading to the prevalence of a proinflammatory and prothrombotic phenotype (4, 5). In these procedures activation of the renin-angiotensin-aldosterone system also holds a central pathogenetic role (6).

Importantly, recent emerging evidence has shown that in EH, an excessive or prolonged stimulation of innate immune cells mediates a chronic inflammatory state that promotes vascular injury and target organ damage (3). Neutrophils represent the most abundant innate immune cells and through the secretion of several prooxidant and proinflammatory molecules are able to promote and facilitate immune-mediated inflammation at sites of tissue injury (7). However, even though epidemiological studies have correlated neutrophil count with increased risk of developing EH and kidney dysfunction (8, 9), the experimental and clinical evidence regarding their exact role in the pathogenesis and complications of EH is limited (10).

Increased attention has been paid recently to neutrophils’ activation in response to a wide variety of stimuli, mainly including microbial factors, ROS, and activated platelets, which leads to the unfurling of their DNA into the extracellular space, thereby forming neutrophil extracellular traps (NETs) and NETosis. NETs constitute a meshwork of extruded chromatin fibers decorated with various highly active neutrophil-derived granular and cytosolic proteins, such as myeloperoxidase (MPO), neutrophil elastase (NE), and citrullinated histones (11, 12). Apart from their protective antimicrobial role, excessive NETs/NETosis may have a detrimental effect to the host by inducing autoimmunity, excessively activating the innate and adaptive immune system, and promoting endothelial damage and inflammation (12, 13). Most importantly, NETs may exert significant prothrombotic properties by presenting extrinsic neutrophil proteins, namely tissue factor (TF), which represents the main in vivo initiator of the coagulation cascade (14, 15). In this context, experimental data in patients with coronary thrombosis have shown that NETs expose a highly functional TF, which is able to induce both thrombin generation and thrombin/protease-activated receptor 1–mediated (PAR1-mediated) platelet activation (16). As such, NETs have been implicated as key drivers in atherothrombosis and atherosclerosis in CVD (14, 17). However, the role of NETs in EH and related complications is largely unknown.

Herein, we identified neutrophils/NETs expressing TF as main components of EH pathogenesis. This study unveils an inflammatory effect of angiotensin II (Ang II) through the induction of ROS/NETosis pathway, leading to thromboinflammation and fibrotic damage in EH.

## Results

### Increased levels of circulating NETs in untreated, newly diagnosed patients with EH.

**Figure 1:**
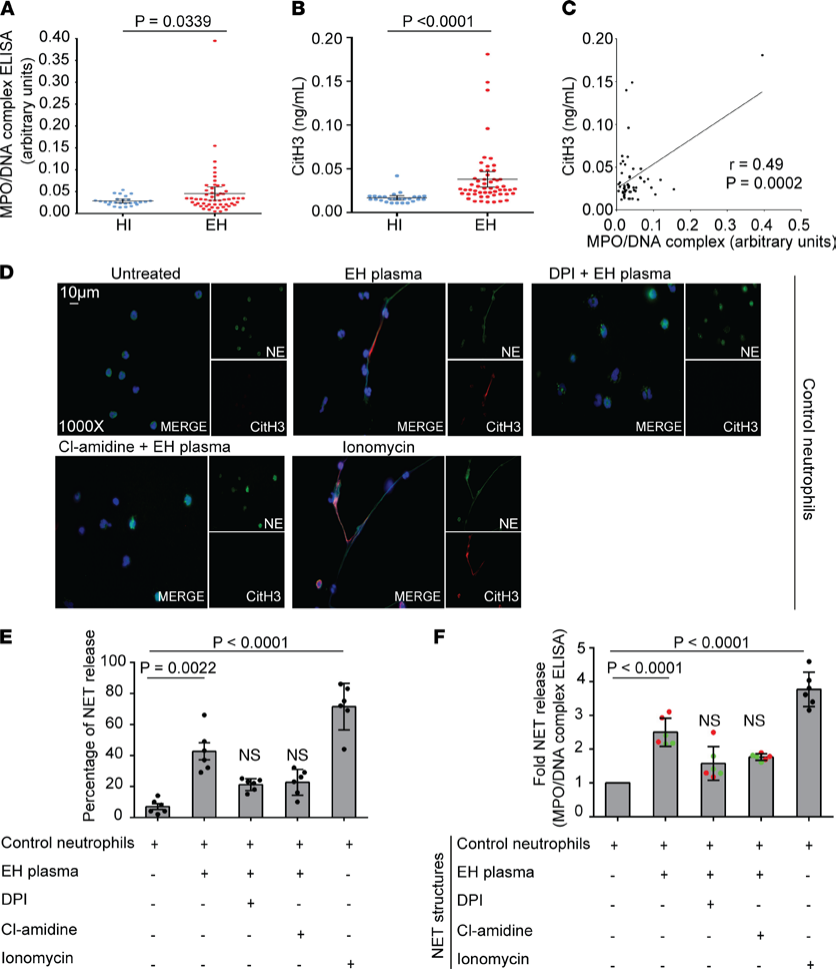
Markers of NETs are detected both in plasma of patients with EH and in control neutrophils treated with EH plasma. (**A**) MPO-DNA complex levels and (**B**) CitH3 levels representing NET release in plasma from healthy individuals (controls, *n* = 26) and patients with EH (*n* = 55). For **A** and **B**, lines represent means accompanied by their ±95% CI, Student’s *t* test (2 tailed). (**C**) Correlation between MPO-DNA levels and CitH3 levels in 2 patients, Pearson’s correlation test. (**D**) Fluorescence microscopy images showing NE/CitH3 staining (blue: DAPI, green: NE, red: CitH3, original magnification, 1000×) and (**E**) percentage of NET release as assessed by immunofluorescence, in control neutrophils incubated with EH plasma and inhibited using diphenyleneiodonium (DPI; NADPH oxidase 2 inhibitor) or Cl-amidine (pan–protein arginine deiminase [pan-PAD] inhibitor). For **D**, a representative example of 6 independent experiments is shown. (**F**) MPO-DNA complex levels in NETs isolated from control neutrophils treated with EH plasma and inhibited with DPI or Cl-amidine. Red and green dots indicate values yielded from control neutrophils that had been incubated with EH plasma samples with higher or lower levels of NET markers, respectively. For **D**–**F**, ionomycin-stimulated neutrophils were used as positive controls. For **E** and **F**, data are from 6 independent experiments (mean ± SD, Friedman’s test). All conditions were compared to controls/untreated (statistically significant: *P* < 0.05; NS: not significant).

Several studies have demonstrated that NETs are implicated in atherosclerosis, fibrosis, and thrombotic CVD (18–20). Thus, we first used ELISA to measure the levels of MPO/DNA complexes and citrullinated histone H3 (CitH3), well-defined circulating markers of NET release (14), in the plasma of EH treatment-naive (untreated) patients. We detected significantly increased levels of these NET remnants in patients compared with healthy individuals (controls) of a similar profile (Figure 1, A and B). Moreover, in patients with EH, CitH3 values were well correlated with MPO/DNA complexes (Figure 1C).

In an effort to support our ex vivo observations, and because distinct inflammatory mediators could drive the formation of NETs (12, 13), neutrophils isolated from healthy individuals (control neutrophils) were stimulated with plasma samples from treatment-naive EH patients that had various levels of NETosis markers (Supplemental Table 1; supplemental material available online; https://doi.org/10.1172/jci.insight.148668DS1). We found that EH plasma–treated control neutrophils generated NETs, as assessed by immunofluorescence microscopy (Figure 1, D and E) and MPO/DNA complex ELISA (Figure 1F). On the other hand, inhibition of NADPH oxidase 2 using DPI or PAD4 using Cl-amidine, prior to stimulation with EH plasma, prevented NET formation, suggesting the involvement of ROS and histone citrullination in this process (Figure 1, D–F).

Collectively, our findings indicate that treatment-naive EH patients exhibited high plasma levels of NETosis, while the inflammatory environment of EH plasma is efficient to induce in vitro NET generation.

### Plasma from patients with untreated EH induces the release of NETs carrying bioactive TF.

**Figure 2:**
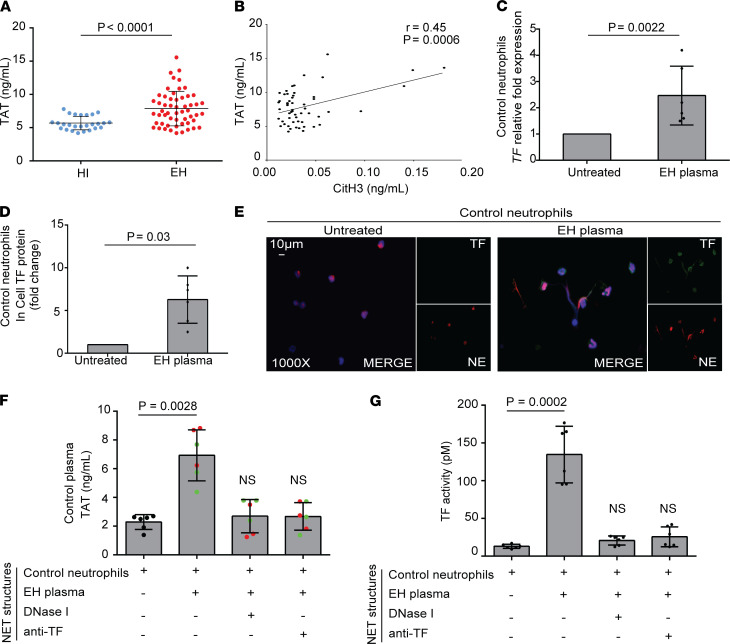
NETs in EH express TF. (**A**) Thrombin-antithrombin (TAT) complex levels in plasma from healthy individuals (controls, *n* = 26) and EH patients (*n* = 55). Lines represent means accompanied by their ±95% CI, Student’s *t* test (2 tailed). (**B**) Correlation between CitH3 representing NET release and TAT levels in EH patients, Pearson’s correlation test. TF expression in control neutrophils treated with EH plasma as assessed by (**C**) qPCR or (**D**) in-cell ELISA. For **C**, *GAPDH* was used to normalize gene expression. For **C** and **D**, Wilcoxon’s test for paired samples was used. (**E**) Fluorescence microscopy images showing TF/NE staining (blue, DAPI; green, TF; red, NE; original magnification, 1000×) in control neutrophils incubated with EH plasma. A representative example of 6 independent experiments is shown. (**F** and **G**) TAT levels and TF activity in in vitro–isolated NET structures, respectively. NETs were obtained by control neutrophils incubated with EH plasma, and subsequently inhibited by DNase I or anti-TF neutralizing antibody, Friedman’s test. In **F**, red and green dots indicate values yielded by incubation of control neutrophils with EH plasma samples that had higher or lower TAT levels, respectively. For **C**, **D**, **F**, and **G**, data are from 6 independent experiments (mean ± SD). All conditions were compared with controls/untreated (statistically significant: *P* < 0.05).

EH is characterized by thrombotic complications. Moreover, NETs have been shown to drive thromboinflammation in several disorders (14, 21, 22). Hence, we measured the levels of thrombin-antithrombin (TAT) complexes in the plasma of patients with untreated EH, and significantly high TAT activity was detected in the patient group compared with controls (Figure 2A), suggesting activation of the TF/thrombin axis in EH. In addition, NET release in plasma, as represented by the levels of CitH3, was significantly correlated with plasma TAT activity, suggesting the thrombogenic potential of NETs in EH (Figure 2B).

To further verify the thromboinflammatory aspect of the disease, in vitro stimulations were deployed. As indicated by TF real-time quantitative PCR (qPCR) and in-cell ELISA, plasma from untreated EH patients was able to induce TF expression in control neutrophils (Figure 2, C and D).

Considering that pathogenesis of neutrophil-mediated diseases is critically defined by the protein composition of NETs (12, 13), next we examined whether TF is externalized on NETs released from control neutrophils upon stimulation with treatment-naive EH plasma samples having various levels of TAT activity (Supplemental Table 1). Indeed, we found that plasma-treated control neutrophils efficiently generated TF-bearing NETs, as assessed by immunofluorescence microscopy (Figure 2E). Importantly, TF on NETs was bioactive, as indicated by TAT ELISA and TF activity quantitative assay (Figure 2, F and G). On the other hand, dismantling NET structures with DNase I or neutralizing TF on NETs significantly reduced TAT levels and TF activity (Figure 2, F and G).

Together, our findings indicate that NETs expressing TF are involved in thrombogenicity of treatment-naive EH patients.

### Ang II induces NET formation in an ROS/autophagy-dependent manner.

**Figure 3:**
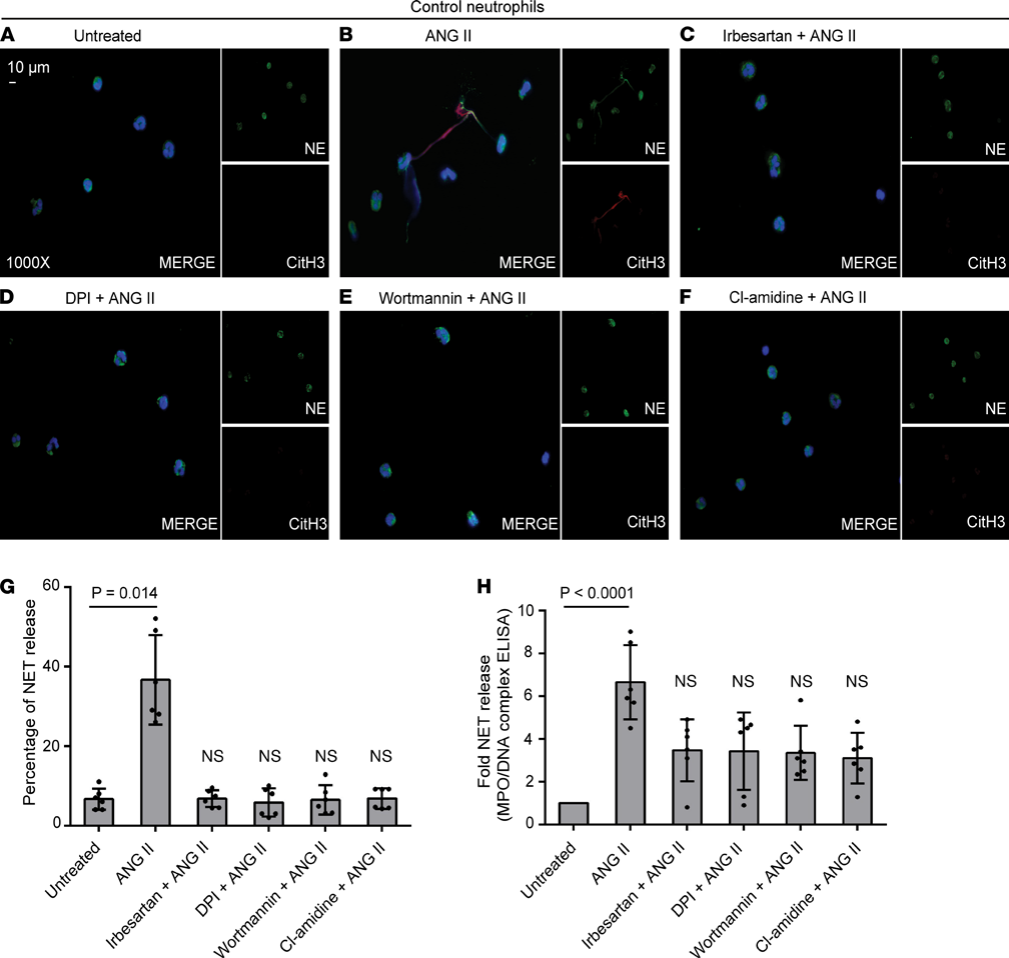
Ang II induces NET formation. (**A**–**F**) Fluorescence microscopy images showing NE/CitH3 staining (blue, DAPI; green, TF; red, NE; original magnification, 1000×) in control neutrophils incubated with 0.1 nM Ang II and inhibited with irbesartan (ARB), DPI (NADPH oxidase 2 inhibitor), wortmannin (early-stage autophagy inhibitor), or Cl-amidine (pan-PAD inhibitor). A representative example of 6 independent experiments is shown. (**G**) Percentage of NET release assessed by immunofluorescence and (**H**) MPO-DNA complex levels in in vitro–isolated NET structures. For **G** and **H**, data are from 6 independent experiments (mean ± SD), Friedman’s test. Inhibitions were performed as described in **A**–**F**. All conditions were compared with untreated (statistically significant: *P* < 0.05).

It has been reported that Ang II exerts an inflammatory effect on human neutrophils, through Ang II type 1 (AT1) receptor inducing neutrophil adhesion and production of ROS (23–26). Hence, we investigated the implication of Ang II in the formation of NETs. Control neutrophils treated with Ang II efficiently generated NETs in a dose-dependent manner (Figure 3, A, B, G, and H, and Supplemental Figure 1A), while Ang II did not exhibit any apoptotic effect on human neutrophils as assessed by annexin V/propidium iodide (PI) staining (Supplemental Figure 1B). On the other hand, pretreatment of cells with irbesartan, an AT1 receptor blocker (ARB), significantly hindered the release of NETs mediated by Ang II (Figure 3, C, G, and H).

Next, we tried to elucidate the possible mechanisms through which Ang II induces NETosis. Distinct mechanisms could mediate and regulate the release of NETs, including ROS, autophagy (27), and PAD4-mediated histone hypercitrullination (12, 27). Hence, prior to stimulation with Ang II, control neutrophils were treated with NADPH oxidase 2 inhibitor DPI, and a significant attenuation of Ang II–mediated NETosis was observed (Figure 3, D, G, and H). Furthermore, to investigate the role of autophagy, cells were preincubated with early-stage (wortmannin) or late-stage (bafilomycin A1, hydroxychloroquine [HCQ]) autophagy inhibitors. Both wortmannin and bafilomycin A1 markedly reduced NET formation (Figure 3, E, G, and H, and Supplemental Figure 2), albeit not statistically significantly for HCQ (Supplemental Figure 2).

Next, we addressed whether PAD4 citrullination of histones is involved in Ang II–induced NETosis. To this end, control neutrophils were treated with Cl-amidine, prior to incubation with Ang II, and a significant reduction of NET formation was observed (Figure 3, F–H).

Collectively, our findings demonstrate that Ang II enhances the formation of NETs in vitro in an ROS/autophagy-dependent manner, and PAD4 histone citrullination is associated with Ang II–induced NETosis.

### Decreased levels of NETs and TF activity in patients with EH treated with ARBs.

Prompted by our in vitro findings indicating that irbesartan reduces the release of Ang II–mediated NETs, we next performed a paired analysis in plasma derived from 12 EH samples collected from patients just before they started treatment with ARBs and 8 weeks later.

**Figure 4:**
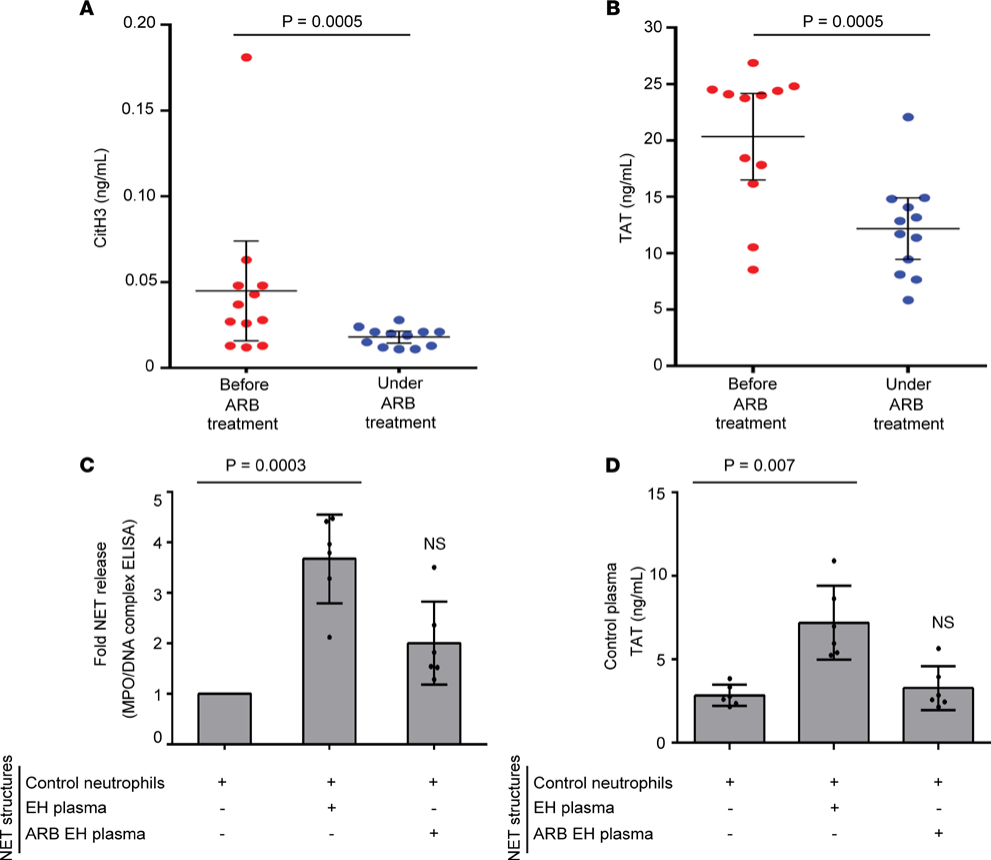
ARBs diminish NETs and TAT activity in EH. Paired analysis of (**A**) CitH3 levels and (**B**) TAT complex levels in plasma, which was obtained from the same EH patients before and under treatment with ARBs (*n* = 12). For **A** and **B**, lines represent means accompanied by their ±95% CI, Wilcoxon’s test for paired samples. (**C**) MPO-DNA complex levels in NETs isolated from control neutrophils stimulated with plasma obtained from EH patients before (EH-plasma) and under treatment with ARB (ARB EH plasma). (**D**) TAT levels in control plasma incubated with NET structures that were isolated from control neutrophils treated with EH plasma or ARB EH plasma, as described in **C**. For **C** and **D**, data are from 6 independent experiments (mean ± SD), Friedman’s test. All conditions were compared with untreated (statistically significant: *P* < 0.05).

Plasma obtained from patients with EH under monotherapy with ARBs (treated EH patients) yielded significantly lower NET release compared with the same patients before the initiation of antihypertensive therapy, as assessed by CitH3 ELISA (Figure 4A). In addition, the subsequent evaluation of TF functionality showed that plasma derived from treated EH patients was characterized by diminished TF activity, as evidenced by TAT assay (Figure 4B).

These results were further reproduced by in vitro stimulations. Control neutrophils incubated with plasma from treated EH patients demonstrated significantly diminished NET formation compared with plasma obtained from the same patients prior to the initiation of ARBs (Figure 4C). Subsequently, the amount of bioactive TF on NETs was found to be reduced in a similar manner, as indicated by TAT ELISA performed on NET structures (Figure 4D).

Taken together, blockage of Ang II signaling is associated with reduced release of NETs and their TF-mediated thrombogenicity in patients with EH.

### TF-bearing NETs activate human endothelial cells toward collagen production.

The association between endothelial dysfunction and EH is well established (28); however, the underlying mechanisms resulting in atherosclerosis are still unclear. In parallel, the crosstalk between NETs and endothelial cells seems to deregulate endothelial function based on previous data (22, 29).

**Figure 5:**
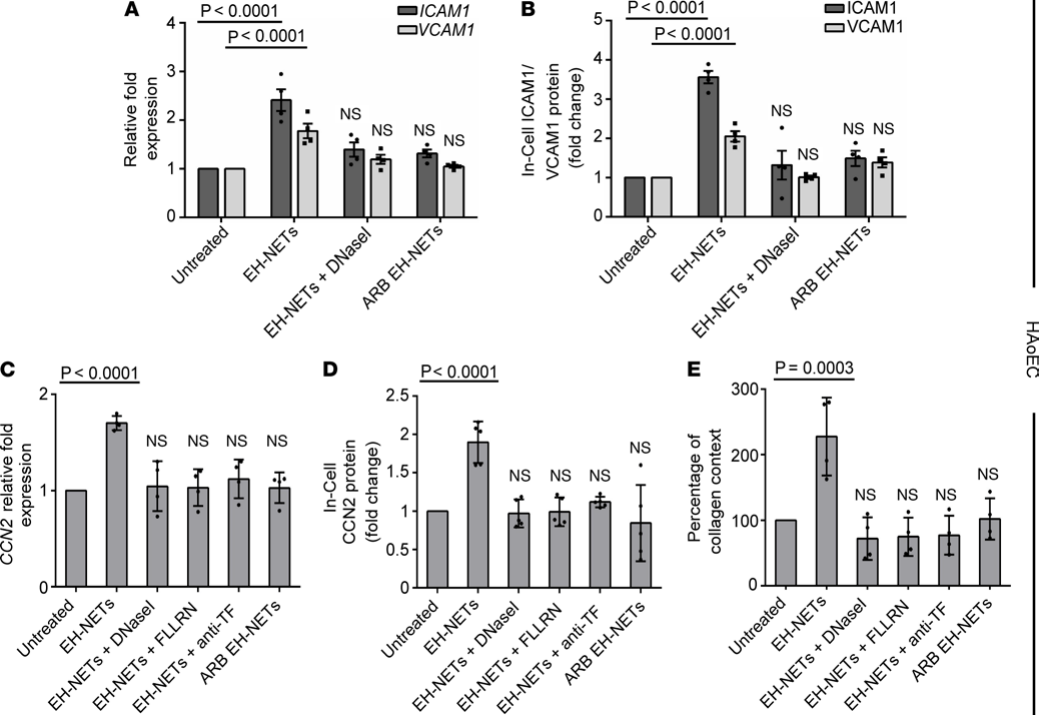
HAoECs acquire a profibrotic phenotype upon incubation with the thromboinflammatory NETs of EH. (**A**) Relative fold expression of mRNA assessed by qPCR and (**B**) surface protein expression assessed by in-cell ELISA for intercellular adhesion molecule 1 (ICAM1) and vascular cell adhesion molecule 1 (VCAM1). HAoECs were incubated with NETs released from neutrophils upon stimulation with plasma from EH patients before (EH-NETs) and after treatment with ARBs (ARB EH-NETs). DNase I was used to dismantle NETs. (**C**) Relative fold expression of mRNA for connective tissue growth factor (CCN2), (**D**) CCN2 protein expression assessed by in-cell ELISA, and (**E**) release of collagen in HAoECs incubated with NETs, as described in **A** and **B**. To hinder TF/thrombin axis, HAoECs were pretreated with FLLRN (PAR1 receptor inhibitor) or EH-NETs preincubated with a neutralizing antibody against TF. For **A** and **C**, *RPL13A* was used to normalize gene expression. For **A**–**E**, data are from 4 independent experiments (mean ± SD), Friedman’s test. All conditions were compared with untreated (statistically significant: *P* < 0.05).

In this context, we deployed a coculture system between human aortic endothelial cells (HAoECs) and disease NETs. As indicated by qPCR and in-cell ELISA, NETs generated in vitro by control neutrophils exposed to plasma from treatment-naive EH patients (hereafter EH-NETs) significantly upregulated endothelial activation markers, compared with untreated HAoECs (Figure 5, A and B). In contrast, NETs generated in vitro by control neutrophils exposed to plasma obtained from patients with EH under ARB treatment (hereafter ARB EH-NETs) were not able to trigger efficient activation of HAoECs (Figure 5, A and B). This was also true, when HAoECs were stimulated with EH-NETs preincubated with DNase I, disrupting the integrity of NET structures (Figure 5, A and B). To further underline the key role of NETs in the activation of HAoECs, NET structures were treated with neutralizing antibodies against main protein components of NETs (12), including cathelicidin LL-37, NE, MPO, or citrullinated histone 4 (H4Cit3). These inhibitions significantly attenuated *ICAM1* and *VCAM1* mRNA expression in HAoECs (Supplemental Figure 3, A and B). These findings suggest that NETs generated in the EH environment may be potent activators of vascular endothelium.

Considering the interplay between NETs and HAoECs’ activation, as well as recent data suggesting that coiled-coil domain containing protein 25 (CCDC25) acts as NET-DNA sensor in cancer cells (30), we investigated whether CCDC25 expression is altered in HAoECs upon stimulation with EH-NETs. As evidenced by qPCR and in-cell ELISA, no significant effect in CCDC25 expression was observed, compared to untreated cells (Supplemental Figure 4, A and B).

Vascular and renal fibrosis are main components of EH-mediated tissue damage. Cellular communication network factor 2 (CCN2) is tightly associated with fibrotic response in various tissues and is a well-known marker of fibrosis (31). Previous evidence has supported that endothelial cells express high levels of CCN2 (32), which is critically involved in the development and progression of atherosclerosis (33). Moreover, endothelial cells can be activated by TF/thrombin pathway through PAR1 receptor (34).

We observed that incubation of HAoECs with NETs generated in vitro by control neutrophils that had been exposed to plasma isolated from treatment-naive EH patients (EH-NETs) significantly enhanced *CCN2* mRNA and protein expression (Figure 5, C and D), as well as collagen production (Figure 5E). This was not true for plasma obtained from ARB-treated patients, which was characterized by diminished levels of NETs (Figure 5, C–E). To highlight the significant role of NET structure integrity on HAoECs’ activity, next EH-NETs were destabilized with DNase I, and a marked decrease of CCN2 expression and collagen release was detected (Figure 5, C–E). To further investigate the interplay between the protein components of NETs and the fibrotic potential of HAoECs, EH-NETs were preincubated with a monoclonal antibody against TF, or HAoECs were pretreated with the FLLRN peptide (PAR-1 receptor blocker) to hinder thrombin signaling. These inhibitions of the TF/thrombin axis resulted in a significant attenuation of endothelial fibrotic markers (Figure 5, C–E), indicating a specific effect of disease TF-bound NETs in HAoEC function.

Together, our findings indicate that bioactive TF on NETs induced by the EH environment may promote endothelial cells’ dysfunction, switching them to a profibrotic phenotype.

### Presence of NETotic neutrophils expressing TF is observed in the fibrotic renal and aneurysmal aortic tissue of patients with EH.

**Figure 6:**
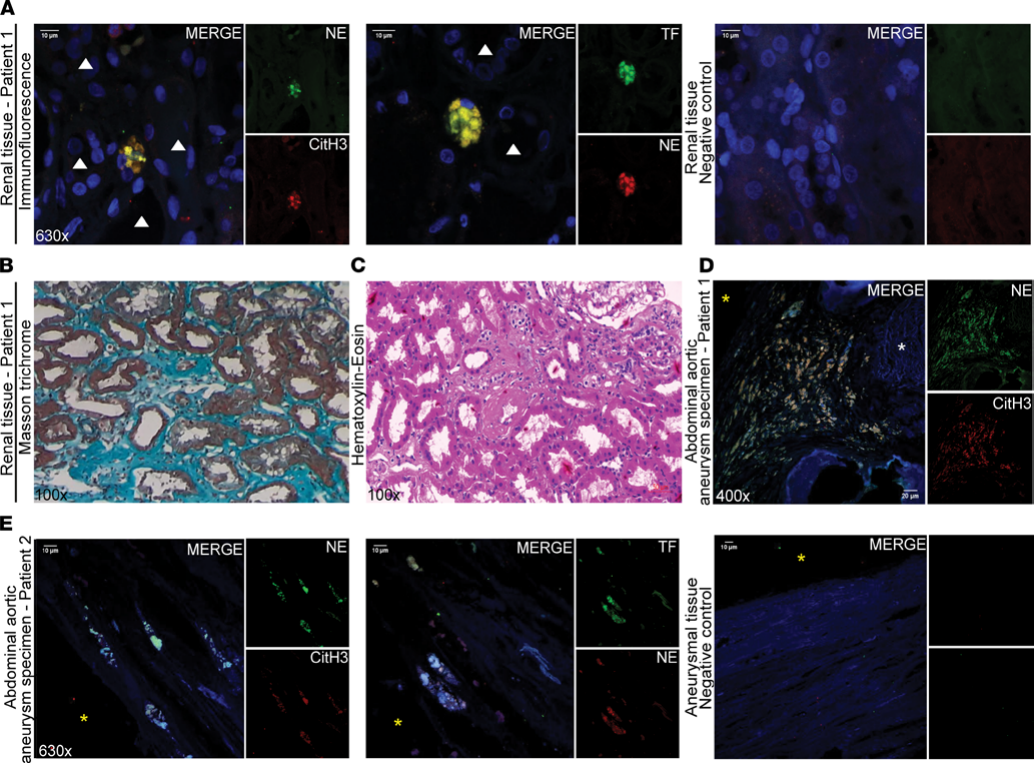
NETotic neutrophils expressing TF are identified in kidney biopsies and AAA specimens from patients with EH. (**A**) NETotic neutrophils/remnants, visualized in renal specimens from a patient with hypertensive nephropathy by costaining with NE and CitH3 (confocal microscopy: blue, DAPI; green, NE; red, CitH3; original magnification, 630×), express TF (confocal microscopy: blue, DAPI; green, TF; red, NE; original magnification, 630×). White arrowheads indicate the renal tubules (either proximal or distant). Renal biopsy is characterized by interstitial fibrosis, as assessed by (**B**) Masson’s trichrome staining and (**C**) hematoxylin & eosin staining (light microscopy, original magnification, 100×). For **A** and **C**, representative data from 1 of 3 patients are shown. (**D** and **E**) NETs were identified in AAA specimens from patients with EH (confocal microscopy: blue, DAPI; green, NE; red, CitH3; original magnification, 400×), bearing TF as indicated in (**E**) (confocal microscopy: blue, DAPI; green, TF; red, NE; original magnification, 630×). For **D** and **E**, representative data from 2 of 3 patients are shown. Yellow asterisk indicates the luminal site of the AAA, and white asterisk indicates disrupted elastic lamina.

To gain further insights into the role of NETs in the pathogenesis of EH, we investigated their presence in kidney biopsies from patients with hypertensive nephropathy and in multiple representative tissue sections of abdominal aortic aneurysm (AAAs) obtained from patients with EH. Of note, we observed NETotic neutrophils and deposition of NET remnants mainly in the renal interstitium, which is commonly characterized by fibrosis in hypertensive nephropathy (35). NETosis was detected as colocalization of NE with CitH3 (Figure 6A), in contrast to their absence in biopsies from normotensive patients suffering from minimal change disease (MCD, Supplemental Figure 5A), which was used as a noninflammatory control renal disorder. Moreover, the NETotic structures in biopsies from patients with hypertensive nephropathy were found to express TF (Figure 6A and Supplemental Figure 5, C and D), in contrast to biopsies from patients with MCD (Supplemental Figure 5B). As expected, patients with hypertensive nephropathy were characterized by renal interstitial fibrosis, as indicated by Masson’s trichrome and hematoxylin & eosin staining (Figure 6, B and C).

NET remnants were also detected in tissue sections from AAA, mainly in the subendothelial layer in the weakened AAA wall in close proximity to the disrupted elastic lamina (Figure 6, D and E). Similar to kidney biopsies, these NETotic remnants were decorated with TF (Figure 6E and Supplemental Figure 5E).

Taken together, the above findings suggest that TF-expressing neutrophils/NETs are present in tissues that are severely affected by EH exhibiting fibrotic and aneurysmal lesions.

## Discussion

This study describes a pathogenic role of Ang II in EH that links neutrophils/NETs with thromboinflammatory tissue injury. The environment of EH triggered the release of NET-bound TF, which exhibited both thrombogenic and profibrotic activity. Ang II emerged as an inducer of the ROS/NETosis pathway promoting collagen production by activated endothelial cells, vascular injury, and interstitial renal fibrosis.

Neutrophils and NETs have been recently recognized as essential players in the initiation and propagation of thromboinflammation in CVDs, such as coronary arterial disease and stroke, which represent well-defined complications of EH (16, 21, 36, 37). We identified that untreated EH patients were characterized by elevated levels of circulating NETs, which were significantly correlated with high thrombogenic plasma activity, as observed by TAT assay. Of note, prothrombotic capacity of EH plasma–induced NETs relied on the delivery of bioactive TF. This is in accordance with recent studies demonstrating EH as a prothrombotic state characterized by increased thrombin generation (5, 38). Moreover, previous research indicated neutrophil functional plasticity leading to TF-bearing NETs as a common pathogenic mechanism in several immunothrombotic conditions (14, 15, 22). Increased NET formation was detected in patients whose cases were newly diagnosed, without any hypertension-related complications, suggesting that thrombogenic NETosis might commence early during EH development. However, these pathologic events subsided after the initiation of antihypertensive treatment with ARBs.

Deregulation of the renin-angiotensin-aldosterone system is thought to be critical for the development of EH; thus, classical antihypertensive treatments target the Ang II axis to effectively control BP (6). In the present study, Ang II was found to be a stimulant of NET formation: while recapitulating our in vitro findings, patients with EH had markedly reduced peripheral blood NETs upon treatment with ARBs, and their plasma abolished the capacity to stimulate procoagulant NET release. Given that EH patients under ARB treatment were normotensive, we cannot exclude that BP control per se could also contribute to these events. Moreover, ARBs such as irbesartan might affect neutrophils through additional to AT1 receptor, pleiotropic modes of action (39). Nevertheless, it appears that Ang II induces NETosis during the EH-related inflammatory response, beyond its classical effects in regulating vascular tone and BP. In a similar way, we have previously described that another BP regulator, epinephrine, is able to stimulate NET formation, linking sympathetic system and adrenergic stress with neutrophil-mediated autoinflammation (40). These results suggest an additional antiinflammatory action for ARBs and further support the association among the neuroendocrine axis, immune system, and stress-induced inflammation (40, 41).

Previous studies have indicated that oxidative stress and autophagy are core and interconnected regulators of NET formation in health and disease (12). Here, we showed that Ang II-mediated NETosis is an ROS-dependent phenomenon, further supporting the role of oxidative stress in EH (42, 43). Furthermore, Ang II-induced NET formation appears to be associated with autophagy machinery and histone citrullination through PAD4. These results are complementary to previous studies demonstrating that Ang II can stimulate neutrophils through AT1 receptors, inducing NADPH oxidase, ROS production, and adhesion to endothelial cells (24, 25). Further studies, which will analyze the Ang II–induced intracellular molecular pathways in depth, may provide additional pieces of the role of Ang II in neutrophils.

Atherosclerosis is characterized by uncontrolled collagen accumulation that leads to arterial stenosis (44). Activated vascular endothelial cells are among cellular populations that contribute to collagen production in atherosclerotic lesions (45). Moreover, atherosclerosis has been associated with NETosis in experimental and human studies (18). This study indicates that thromboinflammatory NETs delivering TF are able to activate in vitro HAoECs, thereby inducing significant CCN2 expression and endothelial collagen production (Figure 5). In a recent COVID-19 study, we found that NET-bound TF activates endothelial cells, increasing their thrombogenicity (22). Here, we further demonstrate a profibrotic effect of the NETs/TF/thrombin axis on endothelium. Taken together and considering our previous work, we propose that TF on NETs may trigger both immediate (immunothrombosis of infection or vascular thrombosis in atherothrombosis) and long-term (vascular fibrosis in atherosclerosis) events. This could be related to the magnitude of inflammatory response (i.e., acute high-grade vs. chronic low-grade) or the total amount of exposed TF. Experiments with DNase I indicated that integrity of NET structure itself may also be crucial for maintaining the bioactivity of TF, as has already been described for other effective NET components in various neutrophil-mediated disorders (12, 13). In a similar way, our previous research showed that NETs promote the in vitro differentiation of mesenchymal cells to collagen-producing myofibroblasts, while NETs and TF/thrombin signaling can accelerate the fibrotic response in patients with systemic autoimmunity (19, 46, 47). Regarding EH, experimental studies have indicated that Ang II promotes vascular fibrosis through direct effects on vascular smooth muscle cells or through the induction of TGF-β and CCN2 (48, 49). This study further suggests a profibrotic role of Ang II by fueling NET-mediated endothelial collagen production.

In order to confirm ex vivo our in vitro observations, we examined biopsies from target tissues affected by long-term EH. Hypertensive nephropathy is among the most important late complications of EH and is frequently characterized by renal interstitial fibrosis directly correlated to progression of CKD (35). Renal interstitial fibrosis is characterized by imbalance between synthesis and degradation of extracellular matrix constituents, leading to excess collagen accumulation (35). Of note, we detected NETotic neutrophils/remnants decorated with TF mainly at sites of interstitial fibrosis. We can assume that TF-bearing NETs facilitate progressively the differentiation of normal resident renal fibroblasts to myofibroblasts, accelerating their fibrotic potential, as already has been described in other clinical disorders (19, 47).

Similarly, we identified NET remnants expressing elastase and TF in the subendothelial layer of AAA tissue specimens obtained from patients suffering long-standing EH. Experimental and clinical evidence have demonstrated that increased turnover and dysfunctional deposition of collagen, in combination with elastin fragmentation, were associated with the onset and progression of AAA (50, 51). Indeed, the observed presence of NETs expressing elastase and TF in the AAA tissue wall supports a pathogenic role of NETosis that probably leads to aneurysmatic vascular remodeling through both elastin degradation (elastase) and collagen production (TF). Accordingly, these results agree with recent data describing an association between inflammatory NETs and AAA formation (52).

In conclusion, this study adds important context regarding the pathogenic role of NETs in EH. Neutrophils activated by the environment of EH expose active TF through NETs. Instigation of the NET/TF/thrombin axis further amplifies the prothrombotic state of EH, promoting vascular damage and renal fibrosis. Mechanistically, Ang II–induced oxidative stress, autophagy, and histone citrullination regulate this axis, suggesting further antiinflammatory and antifibrotic actions for drugs targeting the renin-angiotensin-aldosterone system. Considering that thrombosis and atherosclerosis are largely driven by innate immunity and inflammation, protection from NETosis appears to be an attractive candidate for therapeutic interventions against EH complications.

## Methods

### Patients.

We prospectively recruited 55 adult patients with untreated, newly diagnosed EH and 26 age- and sex-matched healthy individuals (HIs) who served as controls (Supplemental Table 2). Patients with EH were eligible to participate provided they fulfilled the following criteria: (a) age ≥ 18 years; (b) absence of secondary causes of hypertension verified through medical history, physical examination, and appropriate laboratory tests in cases of high suspicion; (c) absence of pregnancy, clinically overt cardiovascular disease, diabetes mellitus, renal disease, and any infectious, inflammatory, or neoplastic disease or other significant comorbidities; (d) absence of current use of antihypertensive or any other cardiovascular medication; (e) no vaccination at least 4 weeks before. Within the group of patients with EH, 12 patients were also evaluated 8 weeks after starting antihypertensive monotherapy with an ARB (irbesartan or olmesartan at maximum doses) who were well controlled in terms of BP. Renal biopsies were retrospectively analyzed from 3 patients with hypertensive nephropathy and 2 normotensive patients with MCD (Supplemental Table 3). Moreover, vascular specimens from AAAs due to hypertension, which were obtained after surgical resection from 3 patients, were prospectively analyzed (Supplemental Table 3).

### Assessment of office BP.

Initially, office BP was measured after 5 minutes in the sitting position using a validated oscillometer device (Microlife Exact BP, Microlife AG) according to the standard recommendations of the European Society of Hypertension (ESH) for office BP measurement (1). The mean of the second and third value of 3 consecutive measurements with a 2-minute interval in the arm with the higher BP was considered as the patient’s office BP.

### Assessment of ambulatory BP.

For all participants, ambulatory BP monitoring was performed in the nondominant arm, with an appropriately sized cuff using the validated Mobil-O-Graph-NG (IEM) device. The device was set to obtain BP values at 20-minute intervals during the day (0700–2259 hours) and at 30-minute intervals during the night period (2300–0659 hours), which were further rearranged according to the actual sleeping hours (from the time the patient went to bed until awaking) reported by each participant. Patients were instructed to maintain their usual activities, avoid strenuous exercise, and keep the arm still at the time of measurements. Measurements were used for the analysis only if more than 70% of the recordings were valid and were analyzed to obtain average 24-hour, daytime, and nighttime systolic BP and diastolic BP values. Hypertension was defined according to the standard ESH guidelines as office BP ≥ 140/90 mmHg and ambulatory daytime BP ≥ 135/85 mmHg (1).

### Laboratory measurements.

For all participants, plasma glucose, lipids (total cholesterol, LDL, HDL, and triglycerides), and kidney function were determined using routine laboratory techniques under 12-hour fasting conditions.

### Serum and plasma collection.

To isolate plasma, venous blood was collected in BD Vacutainer EDTA tubes. Blood was centrifuged at 500*g* for 15 minutes, and then plasma samples were stored at –80°C until analyzed (16, 22).

### Neutrophil isolation.

Peripheral neutrophils were isolated from heparinized blood by double-gradient density centrifugation (11191 and 10771, MilliporeSigma; 30 minutes, 700*g*, at 20°C–25°C) according to the manufacturer’s instructions (53). The cell purity was at least 98%.

### HAoECs culture.

HAoECs were purchased from PromoCell (C-12271). Cells were cultured at 37°C and 5% CO_2_ in endothelial cell growth medium MV2 (C-22020, PromoCell) and passaged after reaching confluence. Cells from passages 3 to 6 were used in the study.

### Stimulation and inhibition studies in peripheral neutrophils.

Neutrophils isolated from HIs (“control neutrophils”) were cultured at 37°C and 5% CO_2_ in RPMI medium (21875, Thermo Fisher Scientific) supplemented with 2% heterologous healthy donor serum. To reproduce the ex vivo findings, control neutrophils were stimulated with plasma derived from EH treatment-naive patients or patients with EΗ treated with ARBs, at a final concentration of 4% in RPMI.

To evaluate the role of Ang II in the release of NETs, control neutrophils were incubated with the peptide hormone (0.1 nM; A9525, Merck KGaA), following appropriate dose experiments (Supplemental Figure 1). To attenuate Ang II signaling, control neutrophils were pretreated (30 minutes) with irbesartan, an ARB (1 μmol/L; I2286, Merck KGaA; ref. 54). To inhibit late-stage autophagy (47), control neutrophils were incubated (30 minutes) with hydroxychloroquine sulfate (HCQ; 50 μM; H0915, Merck KGaA) or bafilomycin A1 (30 nM; SML1661, Merck KGaA). Moreover, neutrophils were treated with wortmannin (100 nM; W3144, Merck KGaA), an early-stage autophagy inhibitor. To hinder ROS production (55), control neutrophils were treated (30 minutes) with DPI (10 μM; D2926, Merck KGaA), an inhibitor of NADPH oxidase. To inhibit PADs, neutrophils were incubated with Cl-amidine (100 μM; 10599, Cayman Chemical). In inhibition studies, neutrophils were pretreated with inhibitory agents for 30 minutes. In in vitro stimulations, control neutrophils were cultured for 3 hours to evaluate NET formation and 90 minutes to study mRNA expression or in-cell ELISA. Ionomycin-treated neutrophils (3 μM; I3909, Merck KGaA) were used as positive control.

### Stimulation and inhibition studies in HAoECs.

To investigate the cross-talk between the TF-bearing NETs and endothelial cells, HAoECs were treated with in vitro–generated NET structures (0.5 μg DNA/mL; ref. 22). To destroy the DNA scaffold, NET structures were incubated with DNase I (1 U/mL; EN0525, Thermo Fisher Scientific), anti-MPO (sc-52707, Santa Cruz Biotechnology Inc), anti-NE (sc-25621, Santa Cruz Biotechnology Inc), anti-H4Cit3 (07-596, Merck KGaA), or anti-LL37 (sc-166770, Santa Cruz Biotechnology Inc), according to manufacturer’s instructions. To hinder PAR1 signaling, HAoECs were treated with the FLLRN peptide (500 μM; AS-60678, Anaspec). To inhibit TF signaling, NETs were treated with an IgG1 goat anti–human TF polyclonal antibody (10 μg/mL; 4501, Sekisui Diagnostics), having a neutralizing effect. In inhibition studies, HAoECs or NET structures were pretreated with the abovementioned inhibitory agents for 30 minutes. For mRNA studies and in-cell ELISA, HAoECs were incubated with NETs for 3 or 6 hours, respectively, at 37°C, 5% CO_2_. For collagen assay, cells were stimulated with NETs for 24 hours, at 37°C, 5% CO_2_, and cell culture supernatants were then collected. The concentrations and time points used to examine neutrophils and HAoECs were optimized before the experiments. All substances used in the study were endotoxin free, as determined by a Limulus amebocyte assay (E8029, MilliporeSigma).

### Ηematoxylin & eosin, Masson’s trichrome, and immunofluorescence staining.

Control neutrophils were seeded onto lysine-coated glass coverslips (Neuvitro; H-12-1.5-PDL), and procedures were performed as previously described (16, 22). In brief, samples were stained using a mouse anti-TF monoclonal antibody (1:200 dilution, sc-59714, monoclonal IgG_1_, Santa Cruz Biotechnology Inc), a rabbit anti-NE polyclonal antibody (1:200 dilution, sc-25621, polyclonal IgG, Santa Cruz Biotechnology Inc), a mouse anti-NE polyclonal antibody (1:200 dilution, sc-55548, polyclonal IgG, Santa Cruz Biotechnology Inc), or a rabbit anti-CitH3 (R2+R8+R17) polyclonal antibody (1:200 dilution, ab5103, polyclonal IgG, Abcam). A rabbit IgG polyclonal antibody (isotype control, ab171870, Abcam) and a mouse IgG polyclonal antibody (isotype control, ab37355, Abcam) were used as controls. DAPI (D9542, MilliporeSigma) was used for DNA staining. Visualization was performed using a fluorescence microscope (OLYMPUS BX51) with a fixed Nikon camera (model DS-Fi1). The percentage of NET-releasing cells was determined by examining 100 cells in a double-blind experimental procedure.

Cross-sections (4 μm thickness) from renal biopsy tissues and mounted whole-tissue sections of abdominal aortic aneurysms were stained with hematoxylin & eosin to assess tissue morphology, using Axio Scan.Z1 (Carl Zeiss) microscope (20×/0.8 Plan-Apochromat objective) and analyzed using ZEN 2.6 Blue software (Zeiss). To evaluate renal fibrosis, Masson’s trichrome staining was further performed in renal biopsies. The histological examination was performed by 2 independent, experienced pathologists. Moreover, all tissue sections were stained with the appropriate antibodies (see above) by double immunofluorescence (19) and visualized using a motorized inverted confocal microscope (Zeiss, LSM710 AxioObserver, Plan-Apochromat 63×/1.40 Oil DIC M27).

### NET isolation.

In brief, 1.5 × 10^6^ purified neutrophils were seeded in 6-well culture plates (Corning), in RPMI culture medium, for 4 hours at 37°C and 5% CO_2_. Following incubation, the culture medium was removed, and each well was washed twice with prewarmed RPMI. To isolate in vitro–generated NET structures, 750 μL of fresh RPMI was added in each well, and NETs adherent to the plate were collected after vigorous agitation. The medium was centrifuged at 20*g* for 5 minutes at 4°C, and supernatant phase, containing NETs, was collected and stored at –20°C until use (16, 56).

### TAT complex ELISA.

The thrombin concentration was measured in (a) EDTA plasma or (b) in vitro–generated NET structures. In the case of NETs, the isolated structures were introduced in healthy plasma at a final concentration of 20%. Then, samples were incubated for 10 minutes at 37°C and immediately transferred on ice, to stop further thrombin activation. The procedure was performed according to the manufacturer’s instructions (ET1020-1, Assaypro) and as previously described (16, 22). Isolated NET structures, treated for 30 minutes with either DNase I (1 U/mL; EN0525, Thermo Fisher Scientific) or anti-TF polyclonal neutralizing antibody (10 μg/mL; 4501, Sekisui Diagnostics), were used as internal controls.

### TF activity assay.

TF activity was measured in in vitro–generated NET structures, using Tissue Factor Human Chromogenic Activity Assay Kit (ab108906, Abcam) in accordance to the manufacturer’s instructions. DNase I (1 U/mL; EN0525, Thermo Fisher Scientific) or anti-TF polyclonal neutralizing antibody (10 μg/mL; 4501, Sekisui Diagnostics) were used as inhibitors.

### In-cell ELISA.

In-cell ELISA was performed in confluent monolayers of (a) HAoECs to measure surface ICAM1, VCAM1, or CCDC25; (b) HAoECs to measure intracellular CCN2; and (c) control neutrophils to measure intracellular TF expression. HAoECs, in 96-well microplates, were incubated with in vitro–generated NET structures for 6 hours. Control neutrophils were stimulated with serum (4% final concentration) derived from EH treatment-naive patients. In all conditions, cells were fixed with 8% paraformaldehyde for 30 minutes. Blocking was performed using 2× blocking solution (ab111541, Abcam) for 2 hours. In the cases of CCN2 (HAoECs cell culture) and TF (neutrophil cell culture), 1× permeabilization buffer was added in cells for 30 minutes. After thorough washing with PBS-1X, cells were incubated with primary anti-human antibodies against ICAM1 (5 μg/mL; ab2213, Abcam), VCAM1 (5 μg/mL; BBA5, Santa Cruz Biotechnology Inc), CCDC25 (5 μg/mL; sc-515201, Santa Cruz Biotechnology Inc), CCN2 (10 μg/mL; sc-14939, Santa Cruz Biotechnology Inc), or TF (10 μg/mL; sc-59714, Santa Cruz Biotechnology Inc) at 4°C overnight. Next, horseradish peroxidase–conjugated rabbit anti–mouse IgG (1:2000 dilution, HAF007, R&D Systems, Bio-Techne), or rabbit anti–goat IgG (1:2000 dilution, HAF109, R&D Systems, Bio-Techne) was added in cells, at room temperature, for 1 hour. After washing with PBS-1X, 100 μL of TMB substrate was added till blue color development. Microplates were measured at 650 nm. The corrections were done by subtracting the signal of the wells incubated in the absence of primary antibody.

### MPO/DNA complex ELISA.

NET-specific MPO-DNA complexes were measured in (a) EDTA plasma or (b) in vitro–generated NET structures. The method was conducted in accordance to the manufacturer’s instructions (Cell Death Detection ELISA Kit, 11544675001, Merck, Kenilworth) and as previously described (57).

### CitH3 ELISA.

CitH3 levels on EDTA plasma were quantified using an H3Cit ELISA kit, according to the instructions of the manufacturer (501620, Cayman Chemical).

### RNA isolation, cDNA synthesis, and qPCR.

Procedures were performed both in neutrophils and in HAoECs, as previously described (16, 22). *GAPDH* or *ribosomal protein L13A* (RPL13A) was used as an internal control gene. Further details regarding the primers and conditions of qPCR are given in Supplemental Table 4. The data were analyzed using the 2^-ΔΔCt^ mathematical model (58).

### Collagen measurement.

The soluble collagen types (I–V) were determined using a Sircol Collagen Assay Kit, according to the instructions of the manufacturer (S1000, Biocolor Ltd). Collagen release was measured in culture supernatants of HAoECs, based on optimization experiments.

### Analysis of neutrophils’ viability.

Apoptosis/necrosis was analyzed in the neutrophil population by flow cytometry (CyFlow Cube 8, PARTEC), as previously described (19). In brief, neutrophils were stained with an FITC-annexin V antibody (an apoptotic marker, BD Biosciences) and PI (a late apoptotic/necrotic marker, MilliporeSigma), after 2 hours of treatment with Ang II. Data were analyzed by FCS Express 4 Flow Cytometry software (De Novo Software).

### Statistics.

Univariate comparisons between patients with EH and HIs (controls) were performed with the use of Pearson’s χ^2^ or a 2-sample independent *t* test (Student’s *t* test, 2 tailed) in cases of categorical and continuous variables, respectively. To check the homogeneity of variance, we used Levene’s test. The nonparametric Wilcoxon test for paired samples was used to compare 2 groups. For comparisons among more groups, the nonparametric Friedman’s test was used. Bivariate correlation analysis was performed using Pearson’s correlation coefficient test (at 95% confidence intervals). The level of statistical significance was set to *P* = 0.05. Means are accompanied by their 95% CIs, unless otherwise indicated. Statistical analysis was performed using GraphPad Prism 6 and SPSS 26.

### Study approval.

The study protocol design was approved by the Local Scientific and Ethics Committees of the University Hospital of Alexandroupolis, and Papageorgiou Hospital of Thessaloniki, Greece. All subjects provided written informed consent in accordance with the principles expressed in the Declaration of Helsinki .

## Author contributions

AC drafted the manuscript, designed and conducted experiments, and analyzed data; EG and AL provided clinical samples, analyzed data, and contributed to writing; SA contributed to tissue data analysis and writing; PP provided clinical samples and contributed to tissue data analysis; MN and AM conducted in vitro experiments; C Antoniadou, C Argyriou, and GSG provided clinical specimens and analyzed data; VP reviewed data and performed statistical analysis; AG reviewed tissue specimens and analyzed data; KR contributed to writing and critically reviewed the manuscript; and PS contributed to writing and conceived, designed, and supervised the study. All authors have read and approved the final manuscript.

## Supplementary Material

Supplemental data

## Version 1 07/29/2021

In-Press Preview

## Version 2 09/22/2021

Electronic publication
