# Supplementary material for: Angiotensin II triggers release of neutrophil extracellular traps, linking thromboinflammation with essential hypertension
Source: JCI Insight. 2021 Sep 22;6(18):e148668. doi: 10.1172/jci.insight.148668 (PMC8492353; doi:10.1172/jci.insight.148668)
Supplement: Supplemental data [file jciinsight-6-148668-s143.pdf]

SUPPLEMENTAL MATERIAL

SUPPLEMENTAL FIGURES

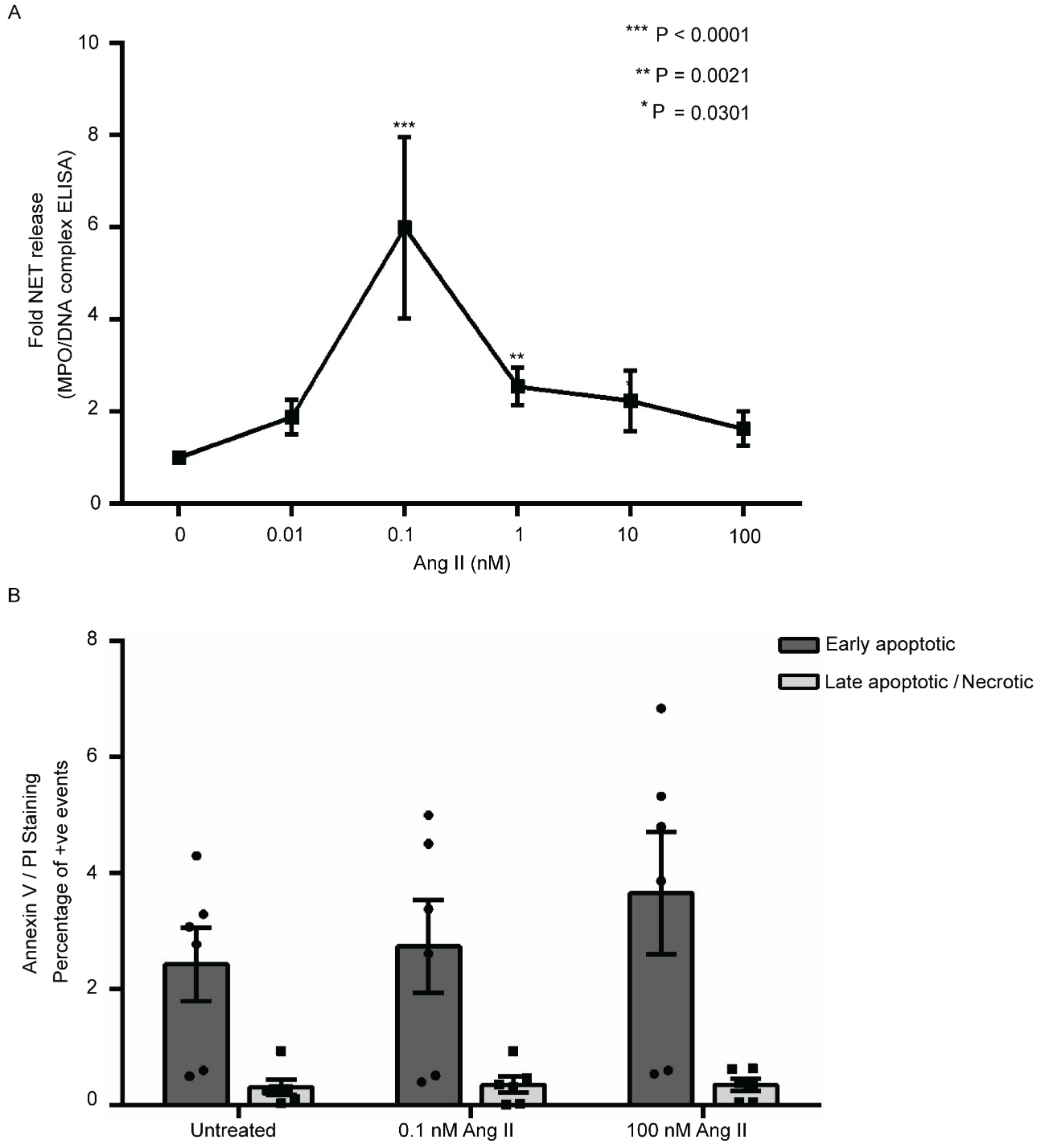

Supplemental Figure S1. A dose-titration study demonstrating that Angiotensin II (Ang II) induces the formation of neutrophil extracellular traps (NETs) ***in vitro*****.** (**A**) Myeloperoxidase (MPO)-DNA complex levels in isolated NET structures, derived from control neutrophils incubated with different doses of Ang II. For titration, a wide range of Ang II doses was used according to Ang II plasma values that have been reported in healthy individuals and pathological conditions, including EH (1–4). Data are from six independent experiments (mean ± SD). (**B**) Early or late apoptosis/necrosis in control neutrophils treated with Ang II (0.1 or 100 nM) for 2h, as assessed by annexin V/PI staining, in flow cytometry analysis. Data are from six independent experiments (mean ± SD). For (A) and (B), Friedman test. All conditions were compared to untreated (statistically significant as p< 0.05, n.s.: not significant).

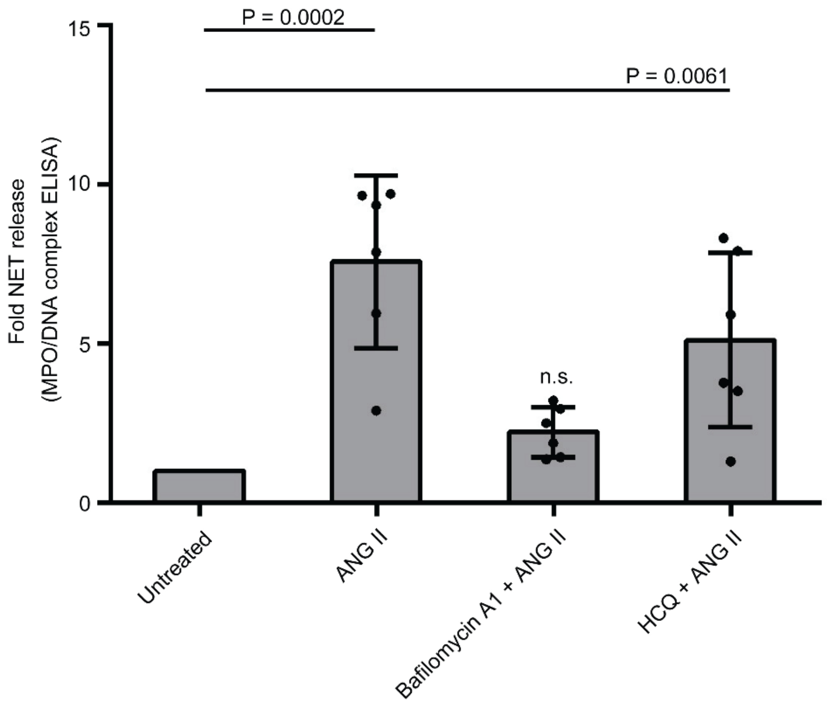

Supplemental Figure S2. Modulation of Angiotensin II (Ang II)-induced NETosis using late-stage autophagy inhibitors. MPO-DNA complex levels in NETs isolated from control neutrophils stimulated with 0.1 nM Ang II and treated with late-stage autophagy inhibitors, i.e. Bafilomycin A1 or hydroxychloroquine (HCQ). Data are from six independent experiments (mean ± SD). All conditions were compared to untreated (statistically significant: $p< 0.05$, n.s.: not significant), Friedman test.

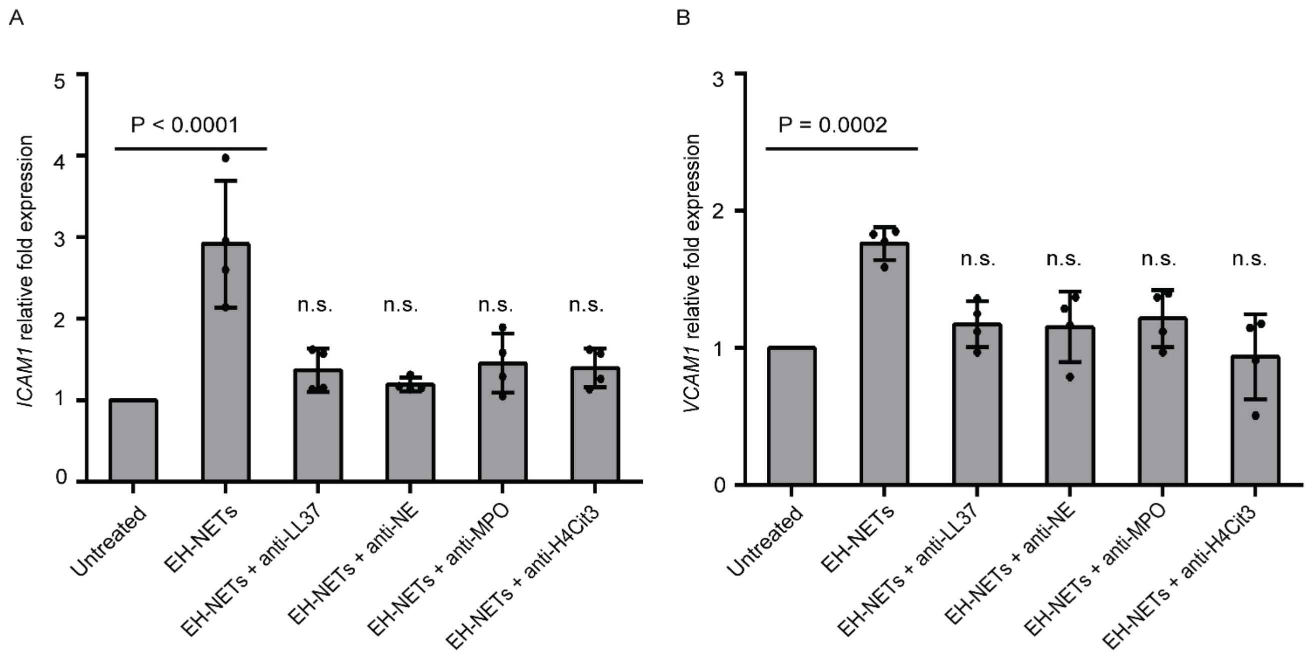

Supplemental Figure S3. The neutralization of NET-components attenuates human aortic endothelial cell (HAoEC) activation. Relative fold expression of mRNA for (**A**) intercellular adhesion molecule 1 (*ICAM1*) and (**B**) vascular cell adhesion molecule 1 (*VCAM1*) in HAoEC incubated with NETs released from control neutrophils treated with plasma from EH patients (EH-NETs). The components of NETs were inhibited using neutralizing antibodies against antimicrobial peptide LL37, neutrophil elastase (NE), myeloperoxidase (MPO) or histone H4 citrulline 3 (H4Cit3). Data are from four independent experiments (mean ± SD). All conditions were compared to untreated (statistically significant: $p< 0.05$, n.s.: not significant), Friedman test.

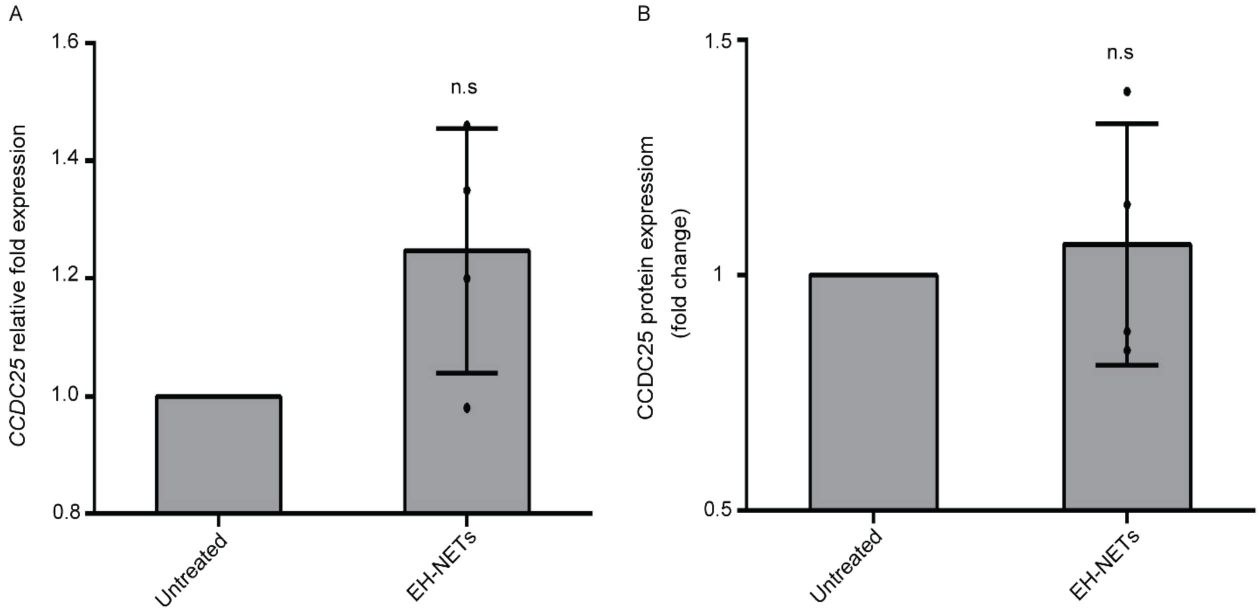

Supplemental Figure S4. The expression of NET-receptor CCDC25 is not up-regulated in human aortic endothelial cells (HAoEC) treated with NETs of essential hypertension (EH). (A) Fold expression of *CCDC25* mRNA in control neutrophils stimulated with NETs released from control neutrophils treated with plasma from EH patients (EH-NETs). *RPL13A* was used to normalize gene expression. (**B**) Surface expression of CCDC25 assessed by In-Cell ELISA in HAoEC treated with EH-NETs. For (A)-(B): Data are from four independent experiments (mean ± SD). All conditions were compared to untreated (statistically significant: $p < 0.05$, n.s.: not significant), Wilcoxon test for paired samples**.**

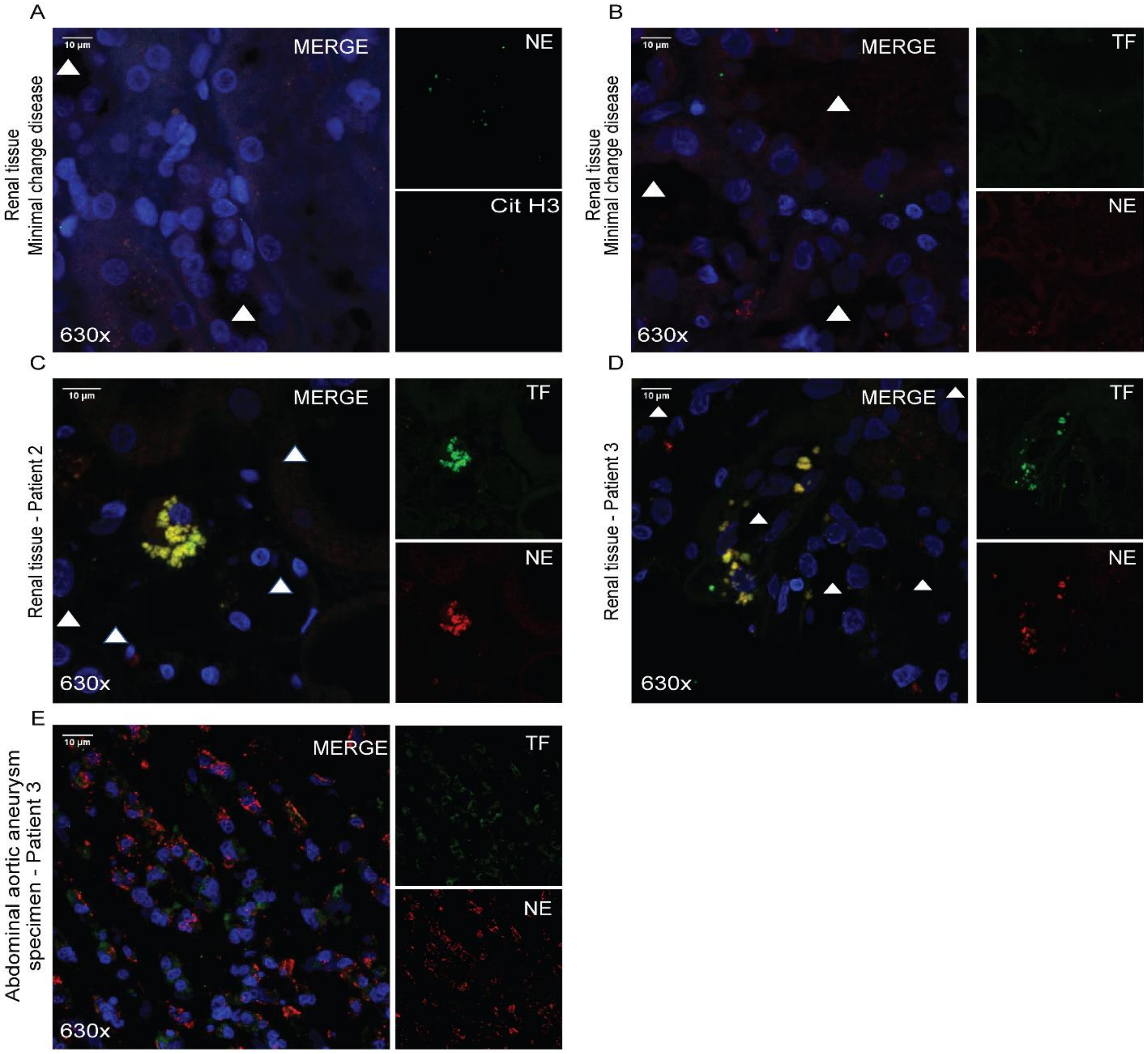

Supplemental Figure S5. NET remnants decorated with tissue factor (TF) are present both in renal biopsies from patients with essential hypertension (EH) and in abdominal aortic aneurysm (AAA) specimens from patients with EH; Absence of NETs in renal specimens from normotensive patients with minimal change disease (MCD). (**A**) Absence of NETotic neutrophils (Confocal microscopy; Blue: DAPI, green: neutrophil elastase/NE, red: citrullinated histone 3/CitH3) or (**B**) NET remnants expressing TF (Confocal microscopy; Blue: DAPI, green: TF, red: neutrophil elastase/NE) in renal specimens from normotensive patients with MCD. Data from one of two patients are shown. (**C**) and (**D**), NETotic neutrophils expressing TF in renal biopsy from patients with hypertensive nephropathy. Representative images from patient 2 and 3 are shown. *White arrowheads* indicate the renal tubules (either proximal or distant). (**E**) Remnants of NETs bearing TF visualized in AAA specimens from patients with EH. Representative image from patient 3 is shown. In (C)-(E), Confocal microscopy; Blue: DAPI, green: TF, red: NE. For (A)-(E), original magnification 630x.

SUPPLEMENTAL TABLES

Supplemental Table S1. EH plasma samples that were used for in vitro stimulations of control neutrophils. Red and green color indicate plasma samples with higher or lower values for NETosis markers (CitH3, MPO/DNA complex) & TAT levels, respectively.

	Values detected ex vivo in EH plasma samples 1-6		
**SAMPLE**	**CitH3 (ng/mL)**	**MPO/DNA complex (AU)**	**TAT (ng/mL)**
**EH plasma _ 1**	0.096	0.065	9.2
**EH plasma _ 2**	0.048	0.035	8.2
**EH plasma _ 3**	0.026	0.015	4.9
**EH plasma _ 4**	0.057	0.053	12.2
**EH plasma _ 5**	0.030	0.017	4.8
**EH plasma _ 6**	0.038	0.032	7.2

Supplemental Table S2. Characteristics and comparison between newly-diagnosed, treatment-naïve essential hypertension (EH) patients and healthy individuals (controls). Statistics: Two-sample independent t-test (continuous variables); Pearson's χ^2 (categorical variables).

**Parameter**	**EH patients (n=55)**	**Healthy Individuals (n=26)**	**P**
Patients characteristics			
Age (years)	48.3±7.7	47.6±8.3	0.712
Male/Female	34 (61.8%)/21 (38.2%)	16 (61.5%)/10 (38.5%)	0.981
Caucasian race	55 (100%)	26 (100%)	1.000
Smoking	24/55 (43.6%)	12/26 (46.2%)	0.831
BMI	29.8±5.3	27.6±4.7	0.067
Blood cells and biochemistry			
Ht (%)	43.4±3.3	43.5±3.3	0.934
Hb (g/dl)	14.6±1.3	14.3±1.1	0.372
MCV (fl)	88.6±5.8	90.0±4.8	0.297
WBC (k/μl)	6.71±1.41	6.48±1.72	0.524
ANC (/μl)	3966±1202	3663±1178	0.290
LNC (/μl)	2093±452	2115±700	0.878
NLR	1.98±0.75	1.89±0.84	0.610
PLT (k/μl)	241±56	247±53	0.643
MPV (fl)	8.24±1.24	7.77±0.70	0.072
Glucose (mg/dl)	87.6±7.8	87.7±7.4	0.968
Creatinine (mg/dl)	0.861±0.174	0.847±0.100	0.644
Uric acid (mg/dl)	5.58±1.42	5.27±1.07	0.331
K^+ (mmol/l)	4.40±0.38	4.37±0.29	0.663
Na^+(mmol/l)	140±2	139±2	0.258
Cholesterol (mg/dl)	208±37	200±33	0.369
Triglycerides (mg/dl)	117±60	113±45	0.734
HDL (mg/dl)	46.4±11.9	47.2±9.9	0.756
LDL (mg/dl)	140±31	131±28	0.210
Microalbumin (mg/24h)	72.4±303	15.6±27.9	0.581
Office BP (mmHg)			
Systolic	154±17	121±11	$<10^{-6}$
Diastolic	100±11	75±9	$<10^{-6}$
ABPM 24h (mmHg)			
Systolic (24h)	146±13	118±6	$<10^{-6}$
Diastolic (24h)	96±10	76±5	$<10^{-6}$
Systolic (day)	149±12	120±8	$<10^{-6}$
Diastolic (day)	99±10	79±4	$<10^{-6}$

BMI: body mass index, Ht: hematocrit, Hb: hemoglobin, MCV: mean corpuscular volume, WBC: white blood cell, ANC: absolute neutrophil count, LNC: absolute lymphocyte count, NLR: neutrophil/lymphocyte ratio, PLT: platelets, MPV: mean platelet volume, HDL: high density lipoprotein, LDL: low density lipoprotein, BP: blood pressure, ABPM: ambulatory blood pressure monitoring

Supplemental Table S3. Characteristics of essential hypertension (EH) patients suffering from hypertensive nephropathy (HN) or abdominal aortic aneurysm (AAA), and patients with minimal change disease (MCD).

**Patients**	**Gender**	**Age (years)**	**EH history (years)**
Hypertensive Nephropathy			
HN-1	Male	40	unknown
HN-2	Male	62	20
HN-3	Male	69	16
Abdominal Aortic Aneurysm			
AAA-1	Male	77	28
AAA-2	Male	85	32
AAA-3	Male	90	24
Minimal Change Disease			
MCD-1	Female	68	No
MCD-2	Female	44	No

Supplemental Table S4. Sequence of Primers [1] and real-time RT-PCR conditions[2]

GENE	PRIMER	SEQUENCE OF PRIMERS	RT-PCR CONDITIONS
VCAM1	FRD:	5’ GACTCCGTCTCATTGACTTGC 3’	1. 52°C for 5 min<br>2. 95°C for 2 min<br>3. 35 cycles of:<br>➢ 95°C for 15 sec<br>➢ 56 ^{O}C for 40 sec<br>4. 52 ^{O}C for 5 min<br>5. Melting curve analysis
	REV:	5’ CATTCGTCACCTTCCCATTCAG 3’	
ICAM1	FRD:	5’ AACCTTCCTCACCGTGTACTG 3’	
	REV:	5’ CTCCACCTGGCAGCGTAG 3’	
CCN2	FRD:	5’ ACCAATGACAACGCCTCCTG 3’	
	REV:	5’ TTGCCCTTCTTAATGTTCTCTTCC 3’	
CCDC25	FRD:	5’ TGAAGATCTGATCAAGCATGG 3’	
	REV:	5’ CAGCACTTCCTTTGGGATGTC 3’	
RPL13A	FRD:	5’ GCCCTACGACAAGAAAAAGCG 3’	
	REV:	5’ TACTTCCAGCCAACCTCGTGA 3’	
TF	FRD:	5’ TTCQGTGTTCAAGCAGTGATTCC 3’	
	REV:	5’ ATGATGACCACAAATACCACAGC 3’	
GAPDH	FRD:	5’ GGGAAGCTTGTCATCAATGG 3’	
	REV:	5’ CATCGCCCCACTTGATTTTG 3’	

[1]Oligonucleotide primers were designed by Beacon Designer™ ver. 4.0.

[2]Real-time PCR was performed using SYBR Green qPCR Master Mix (2x) gene expression master mix (Fermentas, St. Leon-Rot, Germany) on a Chromo4[TM]Real-Time Detector (Bio-Rad, CA, USA).

Supplemental References

1. Schulz A, Jankowski J, Zidek W, Jankowski V. Absolute quantification of endogenous angiotensin II levels in human plasma using ESI-LC-MS/MS. *Clin. Proteomics* 2014;11(1):37.

2. Chu H-T, Li L, Jia M, Diao L-L, Li Z-B. Correlation between serum microRNA-136 levels and RAAS biochemical markers in patients with essential hypertension. *Eur. Rev. Med. Pharmacol. Sci.* 2020;24(22):11761–11767.

3. Roig E et al. Clinical implications of increased plasma angiotensin II despite ACE inhibitor therapy in patients with congestive heart failure. *Eur. Heart J.* 2000;21(1):53–57.

4. Wu Z et al. Elevation of plasma angiotensin II level is a potential pathogenesis for the critically ill COVID-19 patients. *Crit. Care* 2020;24(1):290.
